# How Evolution of Genomes Is Reflected in Exact DNA Sequence Match Statistics

**DOI:** 10.1093/molbev/msu313

**Published:** 2014-11-13

**Authors:** Florian Massip, Michael Sheinman, Sophie Schbath, Peter F. Arndt

**Affiliations:** ^1^Department for Computational Molecular Biology, Max Planck Institute for Molecular Genetics, Ihnestrasse 63–73, 14195 Berlin, Germany; ^2^UR1077, Unite Mathematiques Informatique et Genome, INRA, domaine de Vilvert, Jouy-en-Josas, France

**Keywords:** genome evolution, sequence similarities, segmental duplication, comparative genomics

## Abstract

Genome evolution is shaped by a multitude of mutational processes, including point mutations, insertions, and deletions of DNA sequences, as well as segmental duplications. These mutational processes can leave distinctive qualitative marks in the statistical features of genomic DNA sequences. One such feature is the match length distribution (MLD) of exactly matching sequence segments within an individual genome or between the genomes of related species. These have been observed to exhibit characteristic power law decays in many species. Here, we show that simple dynamical models consisting solely of duplication and mutation processes can already explain the characteristic features of MLDs observed in genomic sequences. Surprisingly, we find that these features are largely insensitive to details of the underlying mutational processes and do not necessarily rely on the action of natural selection. Our results demonstrate how analyzing statistical features of DNA sequences can help us reveal and quantify the different mutational processes that underlie genome evolution.

## Introduction

Genomes evolve in time involving many biological processes that change the heritable information. Although single nucleotide exchanges represent quantitatively the major force, gene duplications and segmental duplications also play a key role in genome evolution (Ohno 1970), especially for innovation purposes. Such DNA duplications in a genome are known for more than 40 years and remain being intensively studied, see for instance Assis and Bachtrog (2013) and Baker et al. (2013). The length of the duplicated sequence segments can range from a few base pairs (for DNA replication slippage), over hundreds of bases (for insertions of repetitive elements or RNA-based duplications), to tens of kilo base pair (for segmental duplications, see Bailey and Eichler 2006 for a review on this topic) or even encompass the whole genome (in the case of a whole-genome duplication).

In this article, we study statistical properties of sequence similarities in a single genome or between two genomes and the different effects of the above-mentioned processes on such similarities. Although segmental duplications generate self-similarities in a single genome, single nucleotide substitutions as well as short insertions and deletions destroy these similarities. The interplay of these processes gives rise to interesting statistical properties as shown below. These properties also prevail after genomes split due to a speciation event. Although in this case segmental duplications do not generate similarities any more, the decay of similarities between genomes features interesting properties due to selective constraints. In regions where selective constraints are important, they slow down the divergence process and maintain similarities between genomes over a longer period of time. Most notably, ultraconserved elements (UCE), which almost do not evolve in time, have been found in many eukaryotic genomes (Bejerano et al. 2004; Reneker et al. 2012).

For our analysis of sequence similarities, we focus on the duplication of DNA segments and mutations. Mutations include single nucleotide substitutions, short insertions, and short deletions. We disregard the so-called repetitive elements, which are small genomic sequences of length ranging from 300 bp to several kilo base pair, able to duplicate themselves many times. They cover a high percentage of many eukaryotic genomes (roughly 50% of the human genome and up to 90% of the maize genome, but only 1.5% of the yeast genome). As they possess their own duplication dynamics, which have already been carefully studied (Cordaux and Batzer 2009), we do not analyze them in this study. For this reason, we analyze eukaryotic genomes where repetitive elements have been masked using the RepeatMasker program (Smit et al. 1996).

As a tool to quantitatively study sequence similarities, we focus on the length distribution of exact matches (segments with an identical sequence) which are maximal, that is, they cannot be extended on either side. Such a match length distribution (MLD) can be obtained for either a self-alignment (by aligning a genome to itself) or for a comparative alignment (by aligning two different genomes to retrieve all maximal exact matches) and will be denoted by *m* in the following. Namely, *m*(*r*) is the number of exact matches of length *r*.

The distribution for a self-alignment of a random sequence or for a comparative alignment of two random sequences of length *L* with the same proportion of the four bases *A*, *C*, *G*, and *T* follows a geometric distribution

(1) $$m_{\text{iid}}(r)∼L^{2}\frac{1}{2}{\left(1-p\right)}^{2}p^{r},$$

where *p* is the probability that two nucleotides match by chance and *r* is the match length. This probability varies with the proportion of each nucleotides in a sequence and is equal to 1/4 if all nucleotides are equally probable. As in a sequence of length L≫r, there are approximately *L* segments of length *r*, the number of expected matches scales as *L*^2^. In the case where *p* = 1/4, we expect less than one match longer than 30 bp in a random genome of 1 Gbp, and about 30 matches of length *r* = 27. In all the distributions, we analyze the following: These so-called random matches always dominate the distribution for small lengths. In this article, we study the behavior observed for longer lengths. In the following, we will refer to this part of the distribution—matches longer than 25 bp, that are not expected to appear in a random sequence—as the tail of the distribution.

**Fig. 1.**
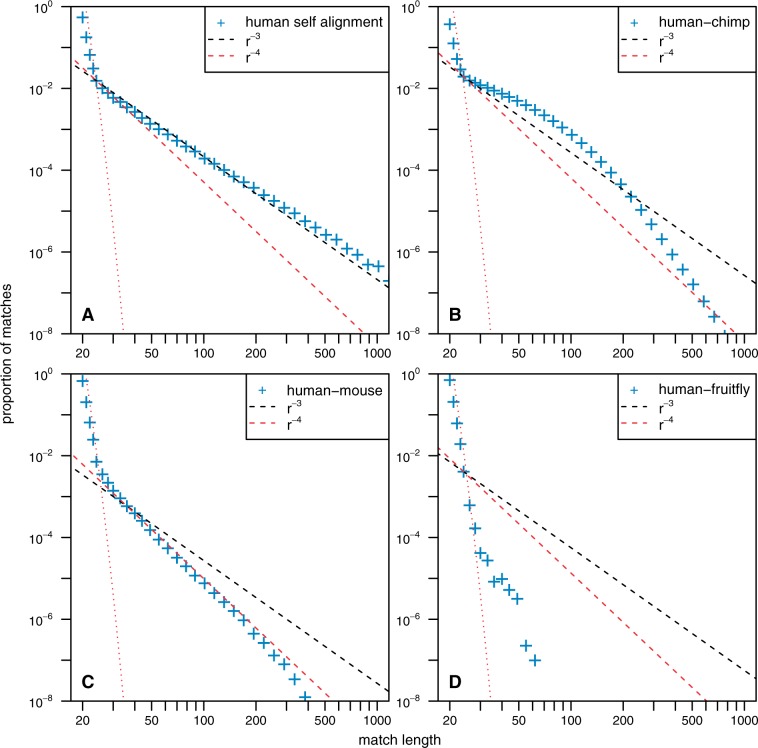
The MLD computed for several genomic alignments involving different species. In all four panels, the red dotted line represent the expected distribution obtained when computing the same experiment on random iid sequences of the same length and the same nucleotide frequencies as the studied species. For small lengths (smaller than 20 bp), MLDs are consistent with these expectations, and we therefore do not show this part in this figure. The dashed line represents power law functions proportional to $1/r^{3}$ (black) and $1/r^{4}$ (red), where *r* is the match length. All empirical data are represented using logarithmic binning to reduce the sampling noise, see Newman (2005) for a discussion on this subject. (*A*) The self-alignment of the repeat-masked version of the human genome. (*B*) The comparative alignment of the human and the chimpanzee genomes, both repeat-masked genomes. (*C*) The comparative alignment of the human and the mouse repeat-masked genomes. (*D*) The comparative alignment of the human and the fruitfly repeat-masked genomes.

Analyzing this distribution for the self-alignment of eukaryotic genomes, an enrichment of long matches has been observed relative to the theoretical distribution $m_{\text{iid}}$ (Gao and Miller 2011). For small matches (smaller than r≃25), the observed distribution follows the theoretical distribution $m_{\text{iid}}$ characterized by an exponential decay, in agreement with equation (1). But fascinatingly, the MLD *m* exhibits a power law tail. Namely, the number of long matches of length *r* scales as

(2) $$m\left(r\right)∼r^{\alpha }$$

with an exponent α≃−3. We reproduce this result for the human genome in figure 1*A*.

Recently, a simple evolutionary neutral model of genome evolution has been proposed by Massip and Arndt (2013). This model includes only random segmental duplications and point mutations and has been demonstrated to generically generate the same statistical property, that is, the power law distribution with exponent α=−3.

However, this model does not explain all observed statistical properties of similarities within and between genomes, since not all of its assumptions are satisfied in the biological context leading to qualitatively different distributions. For instance, we find that the self-alignment of only the retroduplicated part (see Results) of a single genome results in an α=−4 power law distribution of the MLD (fig. 5). Such a distribution cannot be explained using the previously suggested model, which would always result in an α=−3 power law.

Moreover, by comparing the genomes of two distinct species, it was observed that genomes of even evolutionary distant species share many exact matches. Depending on the elapsed time since the divergence of the two species, the length distribution of these maximal exact matches exhibits a different behavior. The MLD computed from two closely related species (say, human and chimpanzee) follows an exponential distribution, while the MLD computed for more distantly related species (say human and mouse) exhibits an α=−4 power law, see figure 1*B* and *C* for an example (Salerno et al. 2006, Gao and Miller 2014). Here, we always refer to the comparison of the human genome against other genomes. Note, however, that we obtained similar results when comparing other pairs of species (for instance, mouse and dog or chimp and rat) at comparable evolutionary distances from each other (see supplementary fig. S5, Supplementary Material online, for some examples).

The previous model ignores that certain genomic regions are conserved and, therefore, does not predict the existence of long sequence matches between genomes of evolutionary distant species. In this study, we extend this model of genome evolution and focus on the consequences of different duplication mechanisms and of sequence conservation due to selective constraints on sequence similarities. In the next section, we show analytically and numerically that different biological processes can account for the described power laws with exponent α=−4 as well as the exponential distribution observed for closely related species.

## Results

**Fig. 2.**
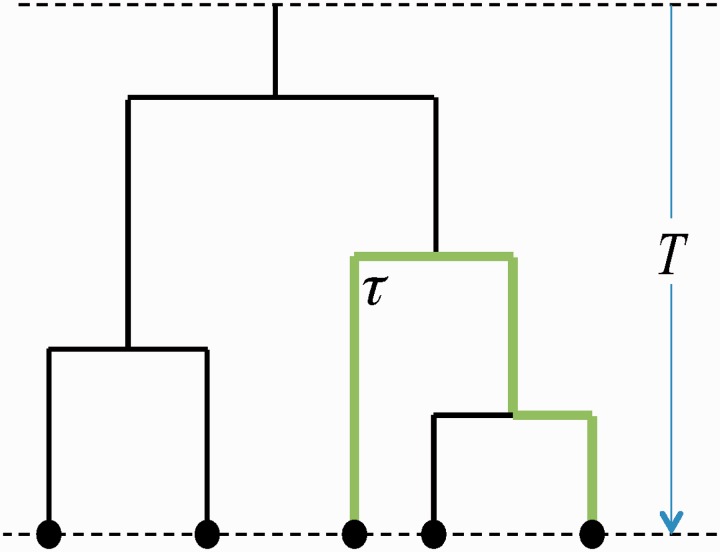
An example of a rooted Yule tree of height *T* with five leaves. The pairwise evolutionary distance between two leaves (green path) is denoted by *τ*. The horizontal dimension is meaningless.

In this section, we analytically calculate the MLDs for different evolutionary processes, and compare them with the distribution observed in real genomes. Let us first focus on the evolutionary fate of one duplicated sequence segment of length *K* under neutral evolution. The duplication generates two identical DNA segments which then evolve independently from each other. In principle, one or both duplicated sequences can duplicate again, giving rise to a branching process where each sequence segment plays the role of a species in a phylogenetic tree. In our framework, the leaves of the tree represent paralogous DNA segments, which share a common ancestor. Any two leaves are separated by some evolutionary time from each other. This dimensionless evolutionary time between a pair of leaves, *τ*, is defined as

(3) $$\tau =\sum_{i}\mu_{i}T_{i},$$

where the sum runs over all the branches along the evolutionary path between the two leaves and *T_i_* and *μ_i_* are the length (in real time) of the branch *i* and the mutation rate (which includes single nucleotide substitutions, short insertions, and deletions) along the branch, respectively (fig. 2).

The number of identical sequence matches of length *r* for a pair of sequences of length *K* separated by a distance *τ* is well described by a random stick-breaking process. Such a process is expected to lead to an exponential tail in the MLD. Indeed, the exact formula is given by

(4) $$m\left(r,\tau \right)=\left[2\tau +\tau^{2}\left(K-r\right)\right]\exp\left(-\tau r\right)$$

for 1≪r<K (see detailed derivation in Ziff and McGrady 1985; Massip and Arndt 2013). Therefore, for a given genome, the match length statistics is obtained by integrating over all pairs of duplicated segments

(5) $$m\left(r\right)=\int_{0}^{\infty }m\left(r,\tau \right)N\left(\tau \right)\text{d}\tau ,$$

where N(τ) is the number of pairs of duplicated sequence segments separated by an evolutionary time *τ*.

In the limit of an infinitely long genome, rare random duplications of sequence segments and the random mutation process yield that N(τ) is constant. These simple assumptions have been shown to give rise to an MLD, *m*, which exhibits a power law distribution $r^{\alpha }$ with α=−3 (Massip and Arndt 2013, Ben-Naim and Krapivsky 2000).

In the following, we relax the above assumptions and calculate N(τ) and the resulting MLD, *m*, for different and biologically more relevant evolutionary scenarios. We start with a scenario where a particular sequence segment and its duplicated offspring duplicate again with a fixed duplication rate. Such a branching process gives rise to a Yule tree (Yule 1925) and we compute the distribution of pairwise distances N(τ) for such trees. A second scenario is meant to represent the retroduplication of an evolutionary well-conserved gene, which gave rise to many pseudogenes during evolution. The resulting tree is clearly different from a Yule tree and the MLD exhibits another power law behavior. We further analyze what happens to the MLD for a comparative alignment of two species that evolve away from their common ancestor.

### Random Segmental Duplication—Yule Trees

We first study the process that gives rise to segmental duplications of DNA segments. According to this process, a segment of length *K* of the genome duplicates again and again with a constant duplication rate per base pair *λ*, such that the duplication rate per segment is *λK*. Each of the resulting segments then have the same duplication rate. The mutation rate *μ* is the same all over the genome. According to this process, one particular segment at time *t* = 0 gives rise to a family of segments. The evolutionary history of such a family can be well described by a Yule tree (fig. 2), and its size grows exponentially in time.

To calculate the theoretical MLD in this Yule tree scenario, we have to compute the distribution of pairwise distances N(τ). Let us focus on the case where we start from one ancestral sequence segment, as exemplified in figure 2. One can derive N(τ) in this case using the following simple arguments. Pairs of leaves, separated by an evolutionary time in the interval [τ,τ+dτ], have branched at the time interval [T−τ/(2μ)−dτ/(2μ),T−τ/(2μ)]. The average number of branching points in this interval is given by the average number of segments at this time, ${\text{e}}^{\lambda K[T-\tau /(2\mu )]}$, times the duplication rate, *λK*, times the length of the interval, dt/(2μ). This results in $\lambda K{\text{e}}^{\lambda K[T-\tau /(2\mu )]}\text{d}t/(2\mu )$. The average number of observed pairs from a branching point in this time interval is given by ${\text{e}}^{2\lambda K\tau /(2\mu )}$. Multiplying the last two factors on obtains the average density of pairs separated by an evolutionary time *τ*:

(6) $$N\left(\tau ,T\right)=\frac{\lambda K}{2\mu }{\text{e}}^{\lambda KT}{\text{e}}^{\lambda K\tau /(2\mu )}$$

for 0≤τ≤2μT and zero otherwise. For a detailed and more general derivation of this and other quantities on Yule trees, see Sheinman M, Massip F, Arndt PF, unpublished data (http://arxiv.org/abs/1407.7821, last accessed July 29, 2014).

Substituting equation (6) in equation (5), one finally obtains for the MLD in the limit rTμ≫1 and λK/(2μ)≪r<K:

(7) $$m\left(r\right)=\frac{\lambda K^{2}{\text{e}}^{\lambda KT}}{\mu }\frac{1}{r^{3}}∼r^{-3}.$$

Interestingly, in this case, the MLD follows the same power law distribution with α=−3 as in the above-mentioned article by Massip and Arndt (2013). In that study, duplications are supposed to occur at random positions and to involve a small fraction of the genome. For this reason, duplications of segments which have already been duplicated are extremely rare, and thus, neglected. In contrast, the so-called Yule model we analyzed in this section takes into account the case where duplicates duplicate again. As shown in the supplementary data, Supplementary Material online, assuming that any segment of the genome can be duplicated, equation (7) becomes

(8) $$m\left(r\right)=\frac{\lambda KL}{\mu }\frac{1}{r^{3}}=A\frac{L}{r^{3}},$$

which is identical to the result obtained by Massip and Arndt (2013). One can derive the value of the longest exact match $r_{\max}$ expected in the neutral case as a function of the prefactor A=λK/μ,

(9) $$r_{\max}≃{\left(AL\right)}^{1/3}$$

if ${\left(AL\right)}^{1/3}\leq K$ and $r_{\max}=K$ otherwise.

Note that using the MLD alone, one cannot distinguish between the two scenarios, in which either all sequence segments duplicate randomly or only a subset of sequences duplicate presumably many times. As the mutation rate is an effective rate subsuming effects of nucleotide exchanges as well as insertion and deletions of short random DNA segments, we also cannot infer the relative contributions of each of these processes.

In figure 1*A*, one can see that these two hypotheses are in good agreement with empirical data from a human genome self-alignment. In supplementary figure S1*B*, Supplementary Material online, we demonstrate that equation (7) is also consistent with numerical simulation of a branching duplication process.

### Retroduplications Generate a Different Pairwise Distance Statistic

Segmental duplication is not the only biological process that produces duplications in eukaryotic genomes. Retroduplication is a well-known biological mechanism which consists of the retrotranscription of an mRNA molecule into the genome. For this reason, retroduplication will solely duplicate transcribed segments of the genome. Besides, this mechanism generates partial duplicates which do not include introns. As retroduplicants also do not contain regulatory elements and promoters, they mostly produce nonfunctional copies, highly similar to the concatenated exons of the functional gene, commonly known as processed pseudogenes (Vanin 1985). Various functions have been found for such pseudogenes, see for instance Kaessmann et al. (2009) or Okamura and Nakai (2008), even though they often result in evolutionary dead ends.

**Fig. 3.**
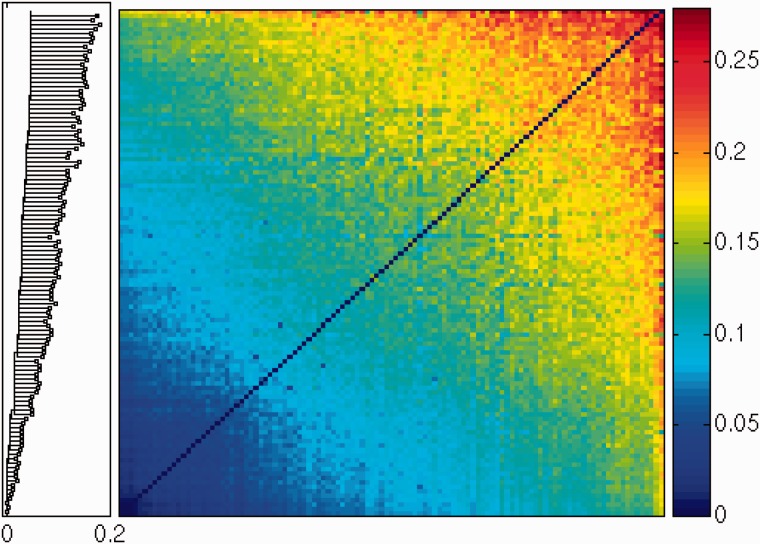
Distance matrix of 113 processed pseudogenes of the RPL21 gene and their phylogenetic tree. The rows and the columns of the distance matrix are sorted with respect to their average. The resulting order is used to constrain the topology of the phylogenetic tree (see Materials and Methods for details).

To study the relationship between the sequences resulting from such process, we studied the large family of 113 processed pseudogenes of the ribosomal protein RPL21 in the human genome. We present the resulting distance matrix and a compatible phylogenetic tree in figure 3 (see Materials and Methods). In contrast to the previous scenario which generates Yule trees, our results on RPL21 suggest that all these pseudogenes were actually generated by retrotranscription of a single functional gene.

**Fig. 4.**
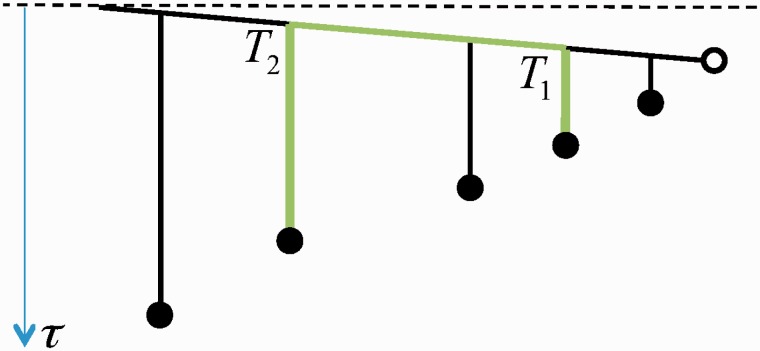
An example of the rooted tree of a pseudogene family (filled circles) stemming from one gene (open circle). The gene evolves much slower than its pseudogenes and the pseudogenes do not retroduplicate. The evolutionary distance between two leaves (green path) is the sum of the evolutionary distance covered by each pseudogene since its retroduplication event and the evolutionary distance covered by the gene between the two retroduplication events. All circles represent contemporary sequence segments.

Following this mechanism, a gene of length *K* duplicates with rate *λK*, while its duplicates (processed, nontranscribed pseudogenes) do not duplicate. Since the evolutionary pressure on the pseudogenes is expected to be much weaker (if any), we assume that the gene and its pseudogenes exhibit different effective mutation rates. This results in a tree similar to the one shown in figure 4.

The evolutionary time that separates two leaves on such a tree is a sum of three times: The evolutionary time elapsed after the first retroduplication event, the evolutionary time elapsed after the second retroduplication event, and the evolutionary time elapsed in the source gene between the two retroduplications (see the green path of the tree in fig. 4). Defining *μ* as the mutation rate of a pseudogene and *μ_S_* as the mutation rate of the source gene, the evolutionary time separating two retroduplicants is given by

(10) $$\tau =\mu \left(T-T_{1}\right)+\mu \left(T-T_{2}\right)+\mu_{S}\left|T_{1}-T_{2}\right|,$$

where *T*_1_ and *T*_2_ are the times at the first and second retroduplications, respectively.

Assuming that *T*_1_ and *T*_2_ are uniformly distributed between 0 and *T*, the density of pseudogene pairs separated by an evolutionary time *τ* after time *T* is given by
(11)
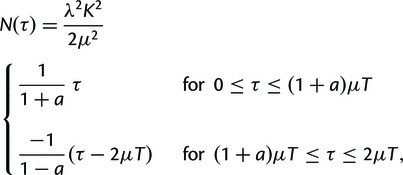

where $a=\mu_{S}/\mu$ and is assumed to be smaller than one, see supplementary data, Supplementary Material online, for calculation details. This is a continuous piecewise linear function, which vanishes for *τ* = 0, namely N(τ=0)=0. It increases linearly with *τ* for small values of *τ*, reaches a maximum at τ=(1+a)μT*,* and then decreases linearly with *τ*, vanishing for τ≥2μT. Such a qualitative trend can be observed in the data for RPL21 pseudogenes shown in figure 3: The number of entries in the distance matrix with small distances is small and increases with the distance, reaches a maximum around 0.12, and then decreases for higher distances.

Substituting equation (11) in equation (5), one obtains in the limit of rTμ≫1 and 0<r≪K the following distribution for the tail of the MLD:

(12) $$m\left(r\right)=\frac{3K^{3}\lambda^{2}}{\left(1+a\right)\mu^{2}}\frac{1}{r^{4}}∼r^{-4},$$

that is, a power law with exponent α=−4. Below we will see that such a power law is generic for distributions of pairs with N(τ=0)=0.

**Fig. 5.**
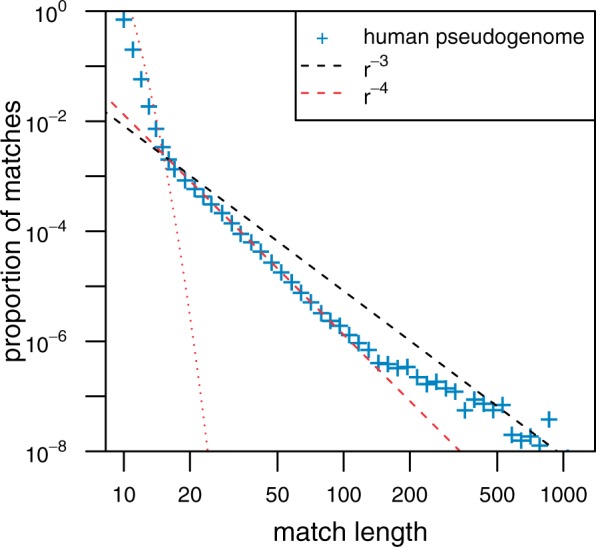
The MLD computed from the self-alignment of the human processed pseudogenome. The total length of this genome is *L* = 6,433,368 bp. The red dotted line represents the expected distribution for random sequences, and the red and black dashed lines represent power laws with exponent α=−4 and α=−3, respectively.

This result also suggests that the self-alignment of processed pseudogenes (retroduplicants) is expected to generate an MLD distributed as a power law with exponent α=−4. Indeed, that is what we observe. We concatenated all the annotated processed pseudogenes of the human genome to construct the so-called human “processed pseudogenome” (see Materials and Methods for more details). The MLD computed from this processed pseudogenome shows a good agreement with our prediction of equation (12) (fig. 5). The deviation of the power law in the very tip of the MLD can be either explained by subsequent segmental duplication of retroduplicated loci or by selective constrains on the retroduplicants making them more conserved than expected by our neutral model.

### Conserved Elements Give Rise to Long Matches in Comparative Alignments of Different Species

As mentioned in the Introduction, one also observes a power law distribution of exact matches between diverged species, as presented in figure 1*C* (see also Salerno et al. 2006). In principle, an RNA-based duplication model described in the previous section could explain the α=−4 power law tail in the MLD of a comparative alignment, if the very same genes are conserved and retroduplicate in both genomes. However, when we compared the two processed pseudogenomes of human and mouse, we found only few exactly conserved sequences and no match longer than 100 bp. Indeed, the sequences of human and mouse homologous genes that have been shown to give rise to many processed pseudogenes, as for instance the RPL21 gene, have already accumulated several independent mutations in the two genomes. For this reason, this process is not responsible for the α=−4 power law observed for the human–mouse comparative MLD.

To definitely rule out the idea that the α=−4 power law observed in the comparative alignment was linked to any duplication mechanism—either RNA or DNA mediated—we filtered out all matches obtained in the human–mouse alignment that were not unique in both genomes (see Materials and Methods for details). Doing so, we filtered out approximately one-third of the matches, but surprisingly, the resulting MLD still exhibits a power law with exponent α=−4 (see supplementary fig. S3, Supplementary Material online). From this experiment, it follows that the orthologous matches are dominant in the human–mouse MLD, and that the power law in this MLD is not the result of any continuous duplication process. This observation leads us to an extension of our model that we present below and for which all matches are unique. In the following, we discuss the properties of comparative alignments and, using a very general set of assumptions, derive the α=−4 power law. We start by describing the MLD just after speciation and then explain how it changes as the divergence between the species gets higher.

Shortly after a speciation event, the genomes of the two resulting species, denoted by *A* and *B*, are almost identical. An alignment of the two genomes will show many long and exact matches, which are either orthologs (along the main diagonal of the alignment grid) or paralogs (off diagonal matches on the alignment grid). The latter are the reminiscences of segmental duplication in the genome of the common ancestor of *A* and *B* and are quantitatively less important than orthologous matches (see previous paragraph). The MLD obtained when comparing these two genomes has always an exponential tail, which stems from orthologous matches. For short evolutionary times, we can assume that mutations happen at random positions along the two genomes and, therefore, the MLD is qualitatively described by the stick breaking model where the initial stick length *K* is now the length of the alignable orthologous part of the two genomes. The tail of such an MLD is therefore exponentially distributed and is given by

(13) $$m\left(r,\tau \right)=\left[2\tau +\tau^{2}\left(K-r\right)\right]\exp\left(-\tau r\right).$$

Indeed, an exponential distribution is also observed in empirical data, for instance for a human–chimp comparison (see fig. 1*B* and Gao and Miller (2014)).

As the divergence increases, the mean length of an observed match decreases fast with time. For this reason, this process alone would not lead to matches of long size in an alignment of genomes of highly divergent species, as, for instance, human and mouse. As the divergence between human and mouse is of the order of 25%, apart from the random matches, we expect only one match of length 72 bp, for aligned genomes of lengths of the order of 1 Gbp (see eq. 13). However, when comparing human and mouse, we obtain 820 exact matches of length 72 bp. Moreover, the MLD observed for human–mouse alignment exhibits a fat tail, shaped as a power law with an exponent α=−4 (fig. 1*C*). This distribution stems from many very well-conserved regions between human and mouse (we obtain more than 6 × 10^5^ exact matches longer than 25 bp, all together they span more than 22 Mbp). Such a power law with an exponent α=−4 has also been observed when comparing other genomes (see supplementary fig. S5, Supplementary Material online).

**Fig. 6.**
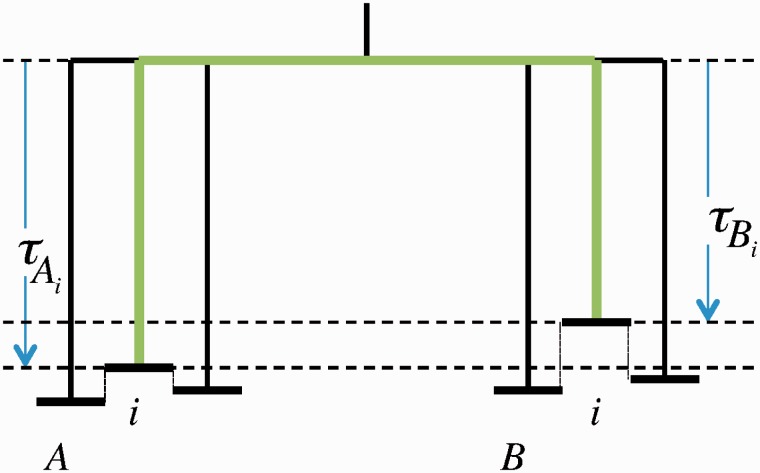
An example of the evolution of two divergent genomes. Different regions of the two species *A* and *B* evolve with different rates. The evolutionary distance separating two orthologous regions *i* (green path) is the sum of the evolutionary distance covered by this genomic region in both species since their split.

If we assume that such a high degree of conservation is the consequence of some biological functionality, it follows that there are regions that evolve at their own (slow) speed, that is, with a lower mutation rate (fig. 6). As each such region can play a different role in the two considered genomes, the mutation rate may be different for the same region in the two different genomes. This leads us to hypothesize that the evolutionary distances between orthologous regions is not constant, but is drawn from some distribution. In the following, we demonstrate that this assumption leads to a qualitative change in the shape of the MLD.

The evolutionary distance between a pair of orthologous sequences is given by

(14) $$\tau =\tau_{A}+\tau_{B},$$

where *τ_A_* is the evolutionary distance from a region in *A* to its orthologous region in the last common ancestor of *A* and *B*, likewise for *τ_B_* (see fig. 6 for an illustration). For a given evolutionary distance, *τ*, the two distances, *τ_A_* and *τ_B_*, can take different values, still satisfying equation (14). The number of sequence regions separated by the evolutionary distance *τ* is therefore given by

(15) $$N(\tau )=\int_{0}^{\tau }N_{A}(\tau -\tau_{B})N_{B}(\tau_{B})\text{d}\tau_{B}$$

where $N_{A}(\tau )$ is the number of sequences in species *A* separated by the evolutionary distance *τ* from its orthologous sequence in the last common ancestor of *A* and *B*, likewise for $N_{B}(\tau )$ (fig. 6).

In general, following equations (4) and (5), the MLD is given by

(16) $$m\left(r\right)=\int_{0}^{\infty }\left[2\tau +\tau^{2}\left(K-r\right)\right]\exp\left(-\tau r\right)N\left(\tau \right)\text{d}\tau .$$

Matches of long length correspond to sequences at small evolutionary distances, *τ*. Thus, the distribution *m* for long length is controlled by the integration over small values of *τ* in equation (16). For such small values of *τ,* the function N(τ) in the integrand of equation (16) can be expanded in a Taylor series around *τ* = 0:

(17) $$N(\tau )=N(\tau =0)+{\frac{\text{d}N(\tau )}{\text{d}\tau }|}_{\tau =0}\tau +O\left(\tau^{2}\right).$$

Equation (15) implies that N(τ=0) always vanishes, such that the next term, $N\prime (0)\tau =N_{A}(0)N_{B}(0)\tau$ linear in *τ*, becomes dominant (see supplementary data, Supplementary Material online). In this case, substituting equation (17) in equation (16) in the regime 1≪r<K results (after integration) in

(18) $$m(r)={\frac{\text{d}N(\tau )}{\text{d}\tau }|}_{\tau =0}\frac{6K-2r}{r^{4}}∼\frac{1}{r^{4}},$$

in agreement with the observed MLD between human and mouse (fig. 1*C*). It follows that the MLD exhibits an α=−4 power law unless the first derivative $\text{d}N(\tau )/\text{d}\tau {|}_{\tau =0}=N_{A}(0)N_{B}(0)$ also vanishes.

Note that a distribution of mutation rates is essential for such a power law to appear. If the mutation rate is not distributed—that is, all regions of the genome have the same mutation rate—then $N_{A}(\tau )$ (resp. $N_{B}(\tau )$) is zero for all values of τ≠μt and thus N(τ)=0 for all τ≠2μt. In this case, the Taylor expansion (17) is not valid and, following equation (16), the MLD is a simple exponential distribution and no power law behavior is expected. In sum, the power law tail with α=−4 in comparative alignment of the genomes indicates that the mutation rate is correlated along any well-conserved DNA region and the distribution of mutation rate is smooth for well-conserved regions and does not vanish at zero.

As mentioned above, in the comparative alignment of any two species, the orthologous sequences (in the diagonal of the alignment grid) are dominating the MLD. One can artificially remove the diagonal part from the alignment. In this case, the remaining paralogous (off-diagonal) DNA segments are expected to exhibit an α=−3 power for closely related species, because in this case the comparative alignment is similar to the self-alignment of one of the species. However, as the divergence between the two species increases, the α=−3 power law is expected to cross over to the α=−4 power law, similarly to the MLD of orthologous sequences, as discussed above. Such a trend was observed recently in Gao and Miller (2014), where off-diagonal alignments were performed for pairs of species of different divergence times.

As shown above, the fact that N(τ=0)=0 in a comparative alignment and the condition $\text{d}N(\tau )/\text{d}\tau {|}_{\tau =0}\neq 0$ result in the α=−4 power law tail for a comparative MLD of distantly related genomes. This condition is indicative for heterogeneity of regional mutation rates along the two genomes. This assumption is quite general and can be fulfilled by a wide range of models (Nei and Kumar 2000). Therefore, the observation of the MLD alone does not allow to decide which of these models describes the actual biological mechanisms responsible for the mutation rate variation.

**Fig. 7.**
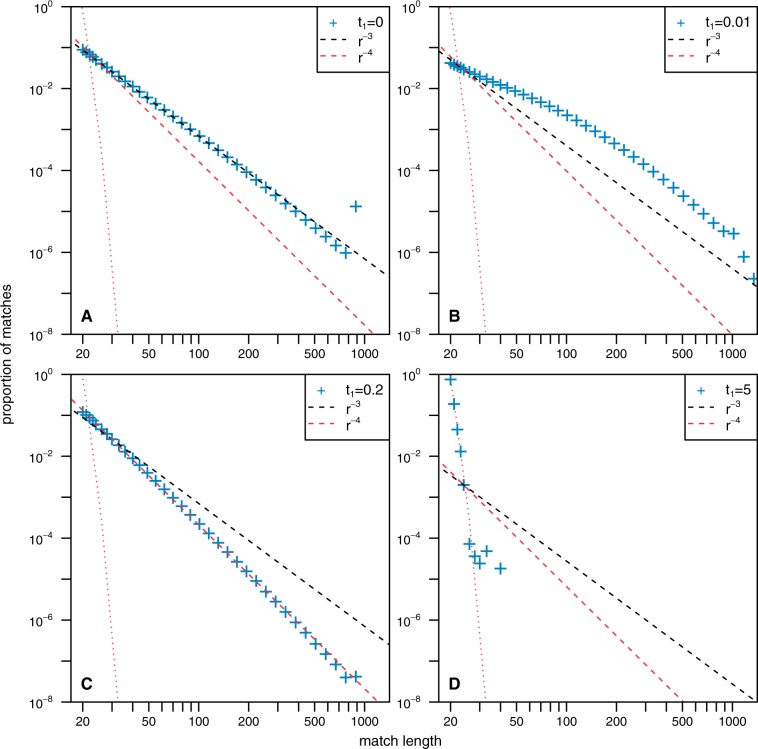
The MLD computed for simulated sequences with various divergence times. In all panels, the red dotted line represents the theoretical distribution obtained when computing the same experiment on random iid sequences with the same length and the same nucleotide frequencies than the simulated sequences. For small lengths, MLDs are consistent with these expectations. The dashed lines represent power law functions proportional to $1/r^{3}$ (black) and $1/r^{4}$ (red), where *r* is the match length. All empirical data are represented using logarithmic binning to reduce the sampling noise. Each plot shows the probability distribution obtained for 10^4^ sequences of length 10^6^ bp. For all simulations, the duplication rate per base pair $\lambda =10^{-3}$, the length of a duplication *K* = 1,000 bp, and the length of a mutating region *M* = 1,000 bp. (*A*) The self-alignment of the common ancestor after $t_{1}=0$. The comparative alignment of two sequences with divergence time $t_{1}=0.01$ (*B*), $t_{1}=0.2$ (*C*), and $t_{1}=5$ (*D*).

In order to illustrate our results, we simulated a model that belongs to this class where $\text{d}N(\tau )/\text{d}\tau {|}_{\tau =0}\neq 0$. For these simulations, we let a synthetic genome evolve according to two simple processes, point mutation and segmental duplication. The genomes are divided into small regions of length *M*, and for each region, we draw a different mutation rate from an exponential distribution with mean 1. We chose the exponential distribution as it is the distribution with minimum information if only the average mutation rate is known. We model both the evolution of one sequence according to this model and the independent evolution of two sequences sharing a common ancestor for various divergence time denoted by *t*_1_ (for details of the simulations, see Materials and Methods). In figure 7, we present the MLD computed from simulated sequences for self-alignment (equivalent to divergence time $t_{1}=0$) and for different divergence times $t_{1}=0.01,t_{1}=0.2$, and $t_{1}=2$. Qualitatively, these simulations exhibit the same behavior as the self-alignment of the human genome, the comparison of human genome with the chimpanzee genome, the comparison of human genome with the mouse genome, and the comparison of human genome with the fruitfly genome, respectively (see also fig. 1 for a comparison).

Note that the mutation rate in this model is constant over small regions of length of the order of the longest expected match (in the simulations we present *M* = 1,000). In the extreme case where the mutation rate is independently chosen for each base pair, that is, with regions of length *M* = 1, we loose this property and shift directly from an exponential distribution for closely related species to no match for distantly related genomes.

## Discussion

In this article, we have shown that only certain evolutionary scenarios are able to account for various empirical power law behaviors in the MLDs of a self-alignment of whole genomes, of processed pseudogenomes, and of the comparative alignment of two distantly related genomes. The basic (and necessary) ingredients for these scenarios are point mutations, duplications, as well as a heterogeneity of mutation rates. Such a heterogeneity reflects the existence of neutrally evolving regions and conserved parts of the genomes, as for instance UCE. For illustrative purposes, we also developed an in silico model of such an evolution and are able to reproduce the empirically observed properties of MLDs in genomes.

**Fig. 8.**
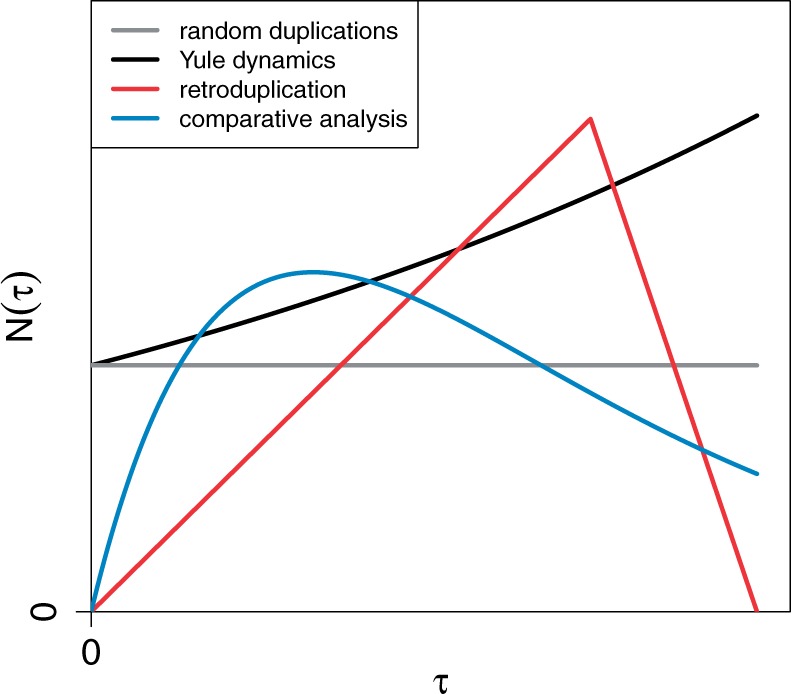
A schematic plot of the functions N(τ) for four different dynamical models of sequence evolution. As explained in the text, different ways of analyzing genomic data (either performing a self-alignment or aligning two genomes) or focusing on distinct compartments (e.g., retro-duplicated pseudogenes) lead to different distributions of pairwise distances between duplicated sequence segments. Only the behavior of N(τ) for small *τ* affects the exponent of the resulting power law *α* in the MLD. If N(τ=0)>0 (e.g., for the first two scenarios), the exponent is α=−3; if N(τ=0)=0, the exponent is α=−4. The functional forms of the first three scenarios are given in the article, the last one is a convolution of two exponential distributions (the exact functional form affects the exponent of the power law tail; see eq. 15).

The exponent of the power law tail of the MLD is determined by the distribution of pairwise evolutionary distances, N(τ)—the number of segments that are at an evolutionary distance equal to *τ* from each other—for small values of *τ*. Above we demonstrate that this function has different shapes for various evolutionary scenarios (see fig. 8 for a summary). The behavior of N(τ) for small values of *τ* is of particular importance: If N(τ=0) is greater than zero, the MLD exhibits an α=−3 power law tail. In the genomic context, this condition implies that segmental duplications occur continuously and therefore homologous pairs of sequences that have not diverged yet exist. However, if N(τ=0) is zero and if the first derivative $\text{d}N(\tau )/\text{d}\tau {|}_{\tau =0}$ is not zero, we expect a power law with exponent α=−4. In the genomic context, the first condition indicates that all homologous sequences have already diverged, and the second one implies that the number of closely related homologous pairs increases linearly with their divergence.

Interestingly, the MLD obtained from the human (or mouse) self-alignment agrees well with an α=−3 power law, indicating that over all processes generating self-similarities in the human (and mouse) genome, the dominant mechanism is the segmental duplication of random sequences of the genome. This observation also implies that random segmental duplications occurred continuously and with a constant rate in the history of these species, and is an ongoing process. If other processes—as for instance retroduplication, whole genome duplication, or burst of segmental duplication—did occur in these genomes, their contribution to the statistical properties of those genomes is negligible compared with random segmental duplications. Note that one cannot judge whether duplicated sequences are prone to duplicate again or not from the knowledge of the MLD alone. In the first case, the duplicated sequences follow a branching process and the Yule framework developed in this article should be used. Otherwise the simple random duplication model introduced by Massip and Arndt (2013) can be used. However, it has been observed that exact matches occurring several times (i.e., more than twice) in the human genome are quite common (Sindi et al. 2008). This observation could be accounted for in the Yule framework, but not with the simple random duplication model where exact matches with more than two occurrences are rare.

Interestingly, we find that the value of the prefactor A=Kλ/μ in equation (8), in genomes exhibiting an α=−3 power law tail, is of the order of 1. Given that the length of these genomes, *L*, is of the order of 1 Gbp and that the typical size of a segmental duplication, *K*, is of the order of 10 kbp, the length of the longest expected exact match is $r_{\max}≃1000$ bp. In a random sequence of the same length, the value of $r_{\max}$ would only be about 30 bp. Note that the value of $r_{\max}$ is not very sensitive to the value of *A* and *L*. For instance, in a genome where *A* = 0.1, the value of $r_{\max}$ would just change 2-fold, resulting in $r_{\max}≃500$.

In contrast to whole-genome self-alignments, we find that if we just concentrate on the human processed pseudogenome, we obtain an α=−4 power law tail. This is due to the different insertion dynamics of processed pseudogenes and the fact that for such a dynamics N(τ) vanishes for small *τ*. The observed α=−4 power law MLD for self-alignment of the whole rabbit genome (see supplementary fig. S2*C*, Supplementary Material online) could therefore also be due to a higher rate of retroduplication in this particular genome. However, many other scenarios can in principle lead to these properties of N(τ) and therefore to an α=−4 power law, as for instance the silencing of the segmental duplication process in recent evolution of a genome, and further analyses would have to be conducted to decide which one is responsible for this behavior in the rabbit genome.

The understanding of MLDs of comparative alignments requires a different reasoning. Due to the evolutionary setup, N(τ=0) always vanishes (see figs. 6 and 8 and eq. 15). We therefore do not expect that the MLD exhibits a power law tail with an exponent α=−3. However, we do expect to observe a power law tail with an exponent α=−4 if the condition $\text{d}N(\tau )/\text{d}\tau {|}_{\tau =0}>0$ is fulfilled. And indeed, the MLD for comparative alignment of human and mouse genomes (as well as for many other pairs) exhibits an α=−4 power law tail. In the text above, we further argue that continuous duplication processes in the two genomes after their split cannot account for the observed power law tail in the MLD. In general, a power law tail can be accounted for by assuming distribution of mutation rates along the genomes, as we have shown analytically and numerically. Surprisingly, for any generic distribution of mutation rates correlated along the genome, the value of *α* is equal to –4, in agreement with empirical observations. This indicates that the mutation rate in the studied genomes fulfils three conditions. First, the mutation rate of well-conserved segments is correlated along the genome with a typical correlation length of at least hundreds of base pairs. Second, there should be nonmutating long regions, such that the distribution of the mutation rate does not vanish at zero. Indeed, comparing eukaryotic genomes numerous such regions have been identified (Bejerano et al. 2004). Third, the mutation rate of well-conserved regions is not the same for all the regions but is continuously distributed. In summary, the distribution of mutation rates of well-conserved regions is a smooth function which does not vanish at zero.

Using similar arguments, one would also expect an α=−4 power law tail to appear in the self-alignment of species which encountered a whole-genome duplication. We observed this behavior for the self-alignment of the genomes of the plant and fish model organisms: *Arabidopsis thaliana* and *Danio rerio* (Zebrafish), in which a whole-genome duplication event occurred recently (Van de Peer 2004; Nakatani et al. 2007; supplementary figs. S2*A* and *B*, Supplementary Material online). However, as stated above, many other duplication scenarios could also lead to an α=−4 power law.

Note that in general, if N(τ) scales as $\tau^{\beta }$ for small values of *τ*, the expected power law is α=−(3+β). Therefore, different integer power laws could be observed if different derivatives in the Taylor expansion of N(τ) vanish. For example, we compared the human (H) and mouse (M) exomes. The resulting MLD exhibits a power law tail with an exponent α=−5 (see supplementary fig. S4, Supplementary Material online), suggesting that in this case the first derivative $N\prime (0)=N_{\text{H}}(0)N_{\text{M}}(0)$ vanishes. This indicates that the distribution of exomic mutation rates vanishes for small rates in at least one of the species, which could be due to relaxed selective constraints on synonymous sites (see supplementary data, Supplementary Material online).

MLDs computed from the self-alignment of many other genomes have been presented by Taillefer and Miller (2014). These MLDs exhibit power laws with various exponent (from α=−2 to α=−4.5). However, genomes with long and highly similar sequences, which are generated by segmental duplications and especially tandem duplications, are not easy to sequence and assemble when using short read next generation sequencing technologies. As the power law behavior only holds for long matches—typically longer than the read length—such power law behavior often remains highly questionable unless the genomic assembly is of a high quality, that is, comparable with the one of the human and mouse genomes. When computing an MLD for a new genome, one would expect to obtain a distribution close to an α=−3 power law. Any deviation from this behavior could in principle be interpreted as a lack of proper repeat masking (notably if one observes peaks for certain lengths in the MLD), a prevalence of another biological process (if one observes a power law with a different exponent) or a poor assembly quality (if one observes a strong deviation from power law behavior). Computing the MLD of a genome, which is a simple and fast computational procedure, can in this sense be of great help in order to understand the biological processes that shape the evolution of this genome and to assess the quality of its assembly.

In conclusion, we have shown that different duplication mechanisms left different footprints in the MLD of eukaryotic genomes. Notably, we have shown that exact self-similarities as long as 1,000 bp in a typical eukaryotic genome could occur without involving any selection. Besides, we have shown that the distribution of matches in a genomic alignment of two species goes through qualitatively different regimes as the genomes diverge (fig. 1). The variance of the mutation rate in different parts of the genomes of the two species guarantees a distribution of identical matches exhibiting power law tail with an exponent α=−4. Such a power law therefore occurs naturally in the MLD of two diverging genomes and is a signature of differences in functional constrains and it is therefore not occurring neutrally.

## Materials and Methods

### Computing Match Length Distributions

To compute the MLD from either a given sequence or two distinct sequences, we first used the MUMmer software to obtain all maximal matches (Kurtz et al. 2004) with the MAXMATCH option to obtain all matches regardless of their uniqueness, and the n option such that the N’s present in the sequences and denoting unknown nucleotide do not match with each other. We then simply counted the resulting number of matches for each length to obtain the MLD.

### Filtering Out Matches with More Than One Occurrence

To rule out the possibility that the α=−4 power law observed in comparative alignments was linked to any duplication mechanism—either RNA or DNA mediated—we filtered out all matches obtained in the human–mouse alignment that were not unique in both genomes. To do so, we first retrieved all the sequences matching in the two genomes (each match between the two genomes corresponds to one sequence). Then, we compared each of these matches against all the other matches using the MUMmer software with MAXMATCH and n options to get all matches longer than 20. Segments that do match with another segment are then considered nonunique. Namely, we define a match as nonunique if it shares a continuous segment of more than 20 bp with any other matches. In supplementary fig. S3, Supplementary Material online, we show that the distribution obtained after filtering out all the matches were not unique in both the mouse and human genome.

#### Numerical Simulations

To simulate the dynamical evolution of a genome under the discussed processes (duplications and mutations) with given rates, we use a Kinetic Monte Carlo scheme.

The first process, mutation, replaces one nucleotide by another one. This process occurs with rate *μ*, which can be understood as the effective mutation rate including insertions and deletions of random sequence segments, because the influence of the latter processes on exactly matching sequence pairs is the same as a nucleotide replacement.

The second process, segmental duplication, occurs with rate *λ* per nucleotide. Depending on the evolutionary scenario, we consider different duplication processes. For “random” segmental duplications, we first choose two random loci, *c* and *v*. Then, a segment of fixed length *K* (we always choose K≪L) starting at position *c* is copied and pasted to the sequence positions starting at *v*. The copied segment replaces the *K* pre-existing nucleotides such that the total length *L* of the sequence remains constant.

If we model the dynamics of segmental duplications following a “Yule process,” we start by duplicating the first *K* nucleotides to the sites adjacent to the right, that is, *c* = 0 and *v* = *K*. The number of duplicated sequence segments is then *n* = 2. For subsequent duplication events, we choose one of the *n* pre-exiting segments and copy it after position *v* = *nK* and increment *n* by one afterwards.

In case we model the dynamics of “retroduplication,” we always duplicate the first *K* nucleotides, that is *c* = 0, and copy them to the positions starting at *v* = *nK*, where *n* is again the total number of duplication events. In this model, we also reduce the rate of nucleotide exchanges for the first *K* positions to mimic the selection on those sites due to functional constrains on a genomic locus.

We start all simulation at *t* = 0 with a random iid sequence of length *L* with all four bases in the same proportions. To generate sequences for self-alignments, we apply the dynamics until a stationary state is reached.

We also use a Kinetic Monte Carlo procedure to generate sequences of species diverging from a common ancestor while including mutation rate heterogeneity. At the beginning of such a simulation, we divide the sequence in different regions of length *M*, such that we have *L/M* regions in total. For each such region, *i*, a mutation rate *μ_i_* is randomly chosen from an exponential distribution with mean 1. This way, some regions are highly conserved (with a low mutation rate), while others evolve fast. In this model, we also include random segmental duplications. We then simulate the dynamics until a stationary state is reached and then duplicate the whole sequence to mimic a speciation event. For each species *A* and *B*, we draw new random mutation rates $\mu_{A_{i}}$ and $\mu_{B_{i}}$ for regions *A_i_* and *B_i_* as above. Later, the dynamics is simulated for some divergence time *t*_1_.The two sequences are aligned to find exactly matching segments.

### Genomic Data

All the repeat-masked genomes we analyze in this article were downloaded from the Ensembl website version 72 (Flicek et al. 2014). For Human, we use GRCh37 release.

### Processed Pseudogenome

To produce the processed pseudogenome, we downloaded the sequence of all 16,889 known pseudogenes of the human genome from the pseudogene.org database. We then filtered these sequences according to their annotation in this database, keeping only those annotated as processed pseudogenes (9,053 pseudogenes left). Using the positions of these different pseudogenes in the genome, we ensure that the different pseudogenes were not overlapping in the human genome. When this was the case (only 25 times), we concatenated the two sequences into one longer sequence containing the two pseudogenes. We then concatenated all the remaining sequences into one long sequence of 6,433 kbp. To separate the different pseudogenes, we added a letter “N” between all sequences. This was done to avoid creating irrelevant matches.

### Phylogenetic Tree of Pseudogenes

To find a set of pseudogenes, we searched for homologous sequences to the RPL21 transcript using BLAST (Altschul et al. 1997). We kept only the sequences with an alignment score larger than half of the length of the RPL21 transcript. This results in 117 sequences. We aligned these sequences using MAFFT program (Katoh and Standley 2013) in the most accurate mode (LINSI). Later, we cleaned the alignment with trimAl (Capella-Gutiérrez et al. 2009) in the automatic mode. To calculate the distance matrix, we used the package PHYLIP (Felsenstein 1989). Four sequences were excluded due to their large distances to other sequences. After calculating the distances, all pseudogenes were ranked according to their average distance to other pseudogenes, from small to large. Then we assumed that the topology of the phylogenetic tree is such that the gene is retroduplicated to the first pseudogene in the ranking and then to the second one, and so on. The tree was built using the same PHYLIP package while the topology of the tree is kept fixed. For this procedure, we used the F84 model (Felsenstein and Churchill 1996) for nucleotides substitutions.

## Supplementary Material

Supplementary figures S1–S5 and Supplementary data are available at *Molecular Biology and Evolution* online (http://www.mbe.oxfordjournals.org/).

Supplementary Data
