# Supplementary material for: How Evolution of Genomes Is Reflected in Exact DNA Sequence Match Statistics
Source: Mol Biol Evol. 2014 Nov 13;32(2):524–35. doi: 10.1093/molbev/msu313 (PMC4298173; doi:10.1093/molbev/msu313)
Supplement: Supplementary Data [file supp_32_2_524__index.html]

How Evolution of Genomes is Reflected in Exact DNA Sequence Match Statistics — How Evolution of Genomes Is Reflected in Exact DNA Sequence Match Statistics — How Evolution of Genomes Is Reflected in Exact DNA Sequence Match Statistics — Supplementary Data 

# How Evolution of Genomes Is Reflected in Exact DNA Sequence Match Statistics

## Supplementary Data

files

**Files in this Data Supplement:**

- Supplementary Data - pdf file
