# Supplementary material for: How Evolution of Genomes Is Reflected in Exact DNA Sequence Match Statistics
Source: Mol Biol Evol. 2014 Nov 13;32(2):524–35. doi: 10.1093/molbev/msu313 (PMC4298173; doi:10.1093/molbev/msu313)
Supplement: Supplementary Data [file supp_msu313_SupplementaryMaterial.pdf]

How Evolution of Genomes is Reflected in Exact DNA Sequence Matches — Supplementary Material —

Florian Massip[†,1,2], Michael Sheinman[†,1], Sophie Schbath[2] and Peter F.Arndt[1]
[1]*Max Planck Institute for Molecular Genetic, Berlin, Germany*
[2]*INRA, UR1077 Unite Mathematique Informatique et Genome, Jouy-en-Josas, France*
[†]*These authors contributed equally*

I. DERIVATION OF EQUATION (8) IN THE MAIN TEXT

Here we explain the derivation of Eq. (8) using Eq. (7). For a single Yule tree, the total average length of the segments after time T is given by $Ke^{\lambda KT}$. If we assume that the genome is composed of n trees, i.e. that there are n families of duplicating non-homologous segments in the genome, the total number of matches in one genome is given by:

$$m(r) = n \ \frac{\lambda K^2 e^{\lambda KT}}{\mu} \frac{1}{r^3}. \tag{S1}$$

The total length of the genome is given by:

$$L = nKe^{\lambda KT}. \tag{S2}$$

Substituting Eq. (S2) in Eq. (S3), we obtain Eq. (8) of the main text, namely:

$$m(r) = \frac{\lambda KL}{\mu} \frac{1}{r^3} = A \frac{L}{r^3}, \tag{S3}$$

with $A = \lambda K/\mu$.

II. PAIRWISE DISTANCE STATISTICS FOR RETRODUPLICATIONS

Consider a DNA segment (coding for a certain mRNA) which is duplicated with rate λ for time T, while its duplicants (processed, non-transcribed pseudogenes) do not duplicate. We assume that, since the evolutionary pressure on the pseudogenes is expected to be weak (if any), the gene and its pseudogenes possess different mutation rates. This results in a tree similar to the one shown in Fig. S1(C).

The evolutionary time that separates two leaves on such a tree is a sum of three times: the evolutionary time elapsed after the first retroduplication event, the evolutionary time elapsed after the second retroduplication event and the evolutionary time elapsed in the source gene between the two retroduplications. Defining μ as the mutation rate of a pseudogene and μ_S as the mutation rate of the source gene, the evolutionary time separating two retroduplicants is given by:

$$\tau = \mu (T - T_1) + \mu (T - T_2) + \mu_S |T_1 - T_2|, \tag{S4}$$

where T_1 and T_2 are the times of the first and second retroduplication, respectively. Assuming a uniform distribution of T_1 and T_2 between 0 and T, the density of pairs of pseudogenes separated by an evolutionary time τ after time T is given then by

$$N(\tau) = \int_0^T \int_0^T \frac{dT_1}{T} \frac{dT_2}{T} \delta \left(\tau - [\mu (T - T_1) + \mu (T - T_2) + \mu_S |T_1 - T_2|]\right), \tag{S5}$$

where δ denotes a Dirac function. This integral is easy to perform and results in Eq. (9).

III. DERIVATION OF THE FIRST DERIVATIVE OF $N(\tau)$ FOR COMPARATIVE ALIGNMENTS

For our comparative analysis we need to compute the Taylor expansion of the function $N(\tau)$ at $\tau = 0$. From Eq. (15) we have:

$$N(\tau) = \int_0^{\tau} N_A(\tau - \tau_B) N_B(\tau_B) d\tau_B. \tag{S6}$$

Using the Leibniz rule to take the derivative of this integral we get

$$\frac{dN(\tau)}{d\tau} = N_A(0)N_B(\tau) + \int_0^{\tau} N'_A(\tau - \tau_B) N_B(\tau_B) d\tau_B, \tag{S7}$$

and thus

$$\left.\frac{dN(\tau)}{d\tau}\right|_{\tau=0} = N_A(0)N_B(0). \tag{S8}$$

Similarly, one can calculate all derivatives of $N(\tau)$. Its Taylor expansion is given by

$$\begin{aligned} N(\tau) \;&=\; 0 + N_A(0)N_B(0)\tau + \left[N'_A(0)N_B(0) + N_A(0)N'_B(0)\right]\frac{\tau^2}{2!} \\ &+ \left[N''_A(0)N_B(0) + N'_A(0)N'_B(0) + N_A(0)N''_B(0)\right]\frac{\tau^3}{3!} + \mathcal{O}(\tau^4). \end{aligned} \tag{S9}$$

Moreover, applying Eqs. (4,5), the MLD is given by

$$\begin{aligned} m(r) \;&=\; 0 \cdot \frac{1}{r^3} + N_A(0)N_B(0)\frac{6(K-r+2)}{r^4} + \left[N'_A(0)N_B(0) + N_A(0)N'_B(0)\right]\frac{12(K-r+2)}{r^5} \\ &+ \left[N''_A(0)N_B(0) + N'_A(0)N'_B(0) + N_A(0)N''_B(0)\right]\frac{20(K-r+2)}{r^6} + ... \end{aligned} \tag{S10}$$

Depending on which term dominates the sum in Eq. (S10), one can predict which power-law tail will be observed in the MLD of the comparative alignment.

In the case of comparative alignment of whole genomes one clearly observe the power-law with an exponent $\alpha = -4$ (see Fig. 1(C)). This indicates that the term $N_A(0)N_B(0)$ is dominant. This is expected because of the existence of ultra-conserved elements in the genomes, such that the distribution of mutation rate does not vanish at zero, giving rise to $N_A(0)N_B(0) > 0$.

The MLD of comparative alignment of human and mouse exomes exhibits a power-law with an exponent $\alpha = -5$ (see Fig. S4). This indicates that in exomes the fraction of non-mutating sequences is small (probably due to relaxed constraints on synonymous sites), such that the term with r^{-5} dominates, resulting in the observed power-law tail.

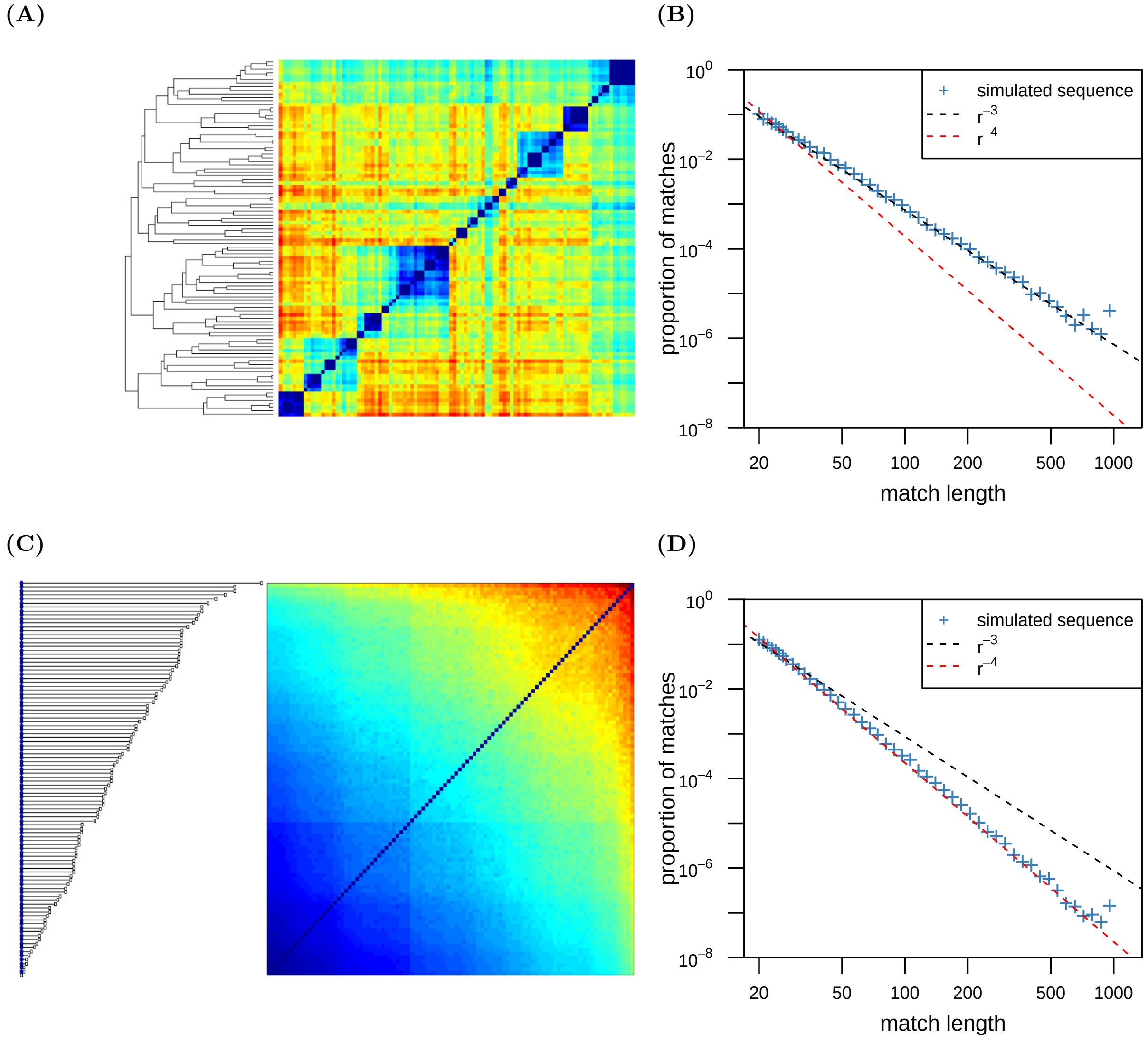

Figure S1: Simulated trees, distance matrices and MLDs for different duplication processes. (A) Tree and distance matrix of a family of sequences for the case where all sequences duplicate with the same duplication rate, λ, and mutate with the same rate, μ, giving rise to a Yule Tree. (B) MLD for the self-alignment of a sequence consisting in the concatenation of all the leaves produced using the process presented in (A). Dashed lines represent power-laws with exponent $\alpha = -4$ (red) and $\alpha = -3$ (black). (C) Corresponding data for a family of pseudogenes where all duplicates stem from the same gene. The duplicated pseudogenes are not functional, and do not duplicate. They accumulate random mutations with rate μ while the source gene has a lower mutation rate, μ_S, taken to be zero for this particular example for simplicity. (D) The corresponding MLD for the self-alignment of a sequence consisting in the concatenation of all the leaves produced using the process presented in (C).

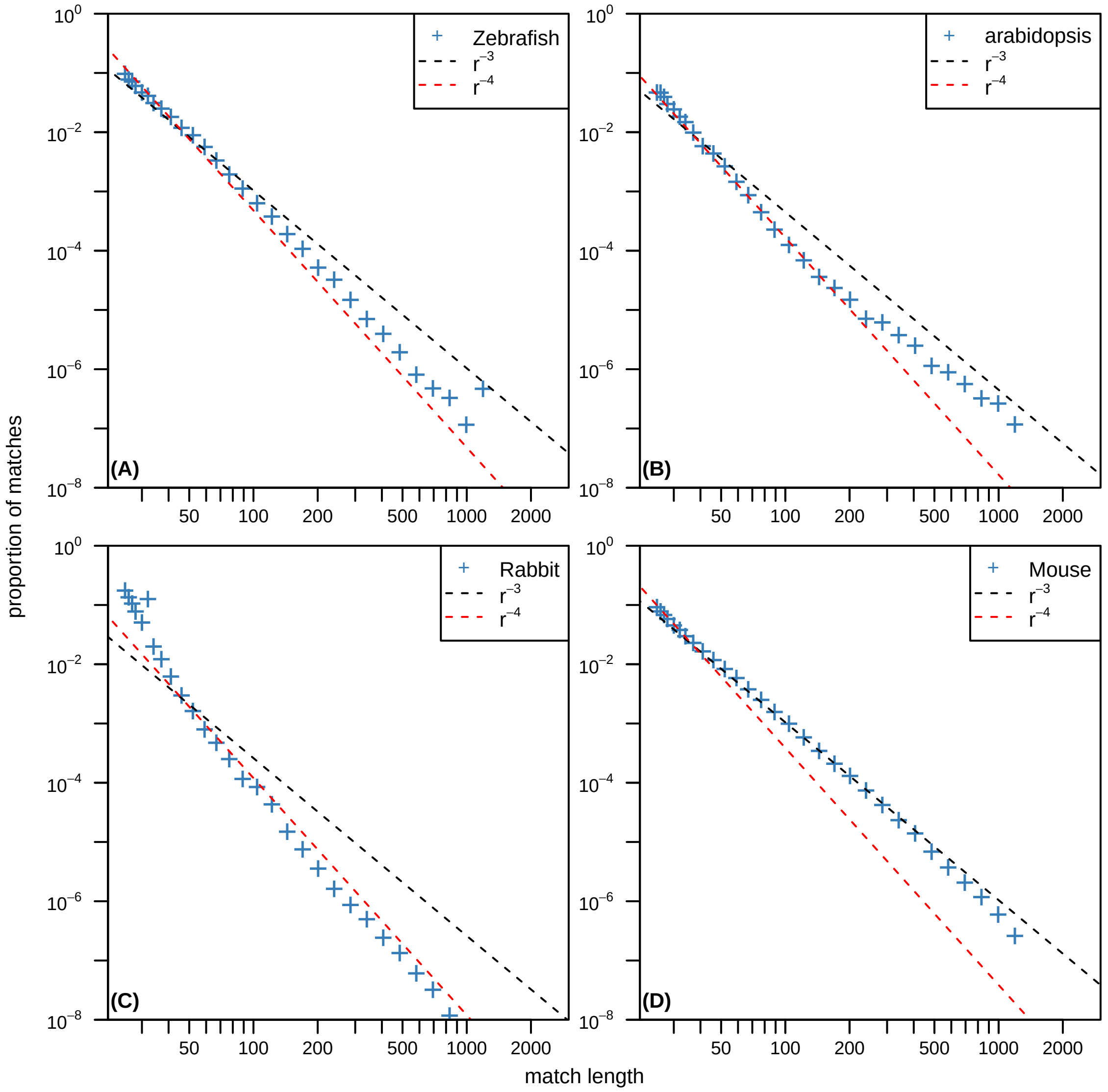

Figure S2: The MLD computed for the self-alignment of RepeatMasked genomes of different species. The dashed lines represent power-laws with exponent $\alpha = -3$ and $\alpha = -4$. All empirical data are represented using logarithmic binning to reduce the sampling noise. (A) The self-alignment of *arabidospis thaliana* genome. (B) The self-alignment of the Zebbrafish (*danio rerio*) genome. (C) The self-alignment of the Rabbit (*oryctolagus cuniculus*) genome. (D) The self-alignment of the Mouse (*mus musculus*) genome excluding the Y chromosome.

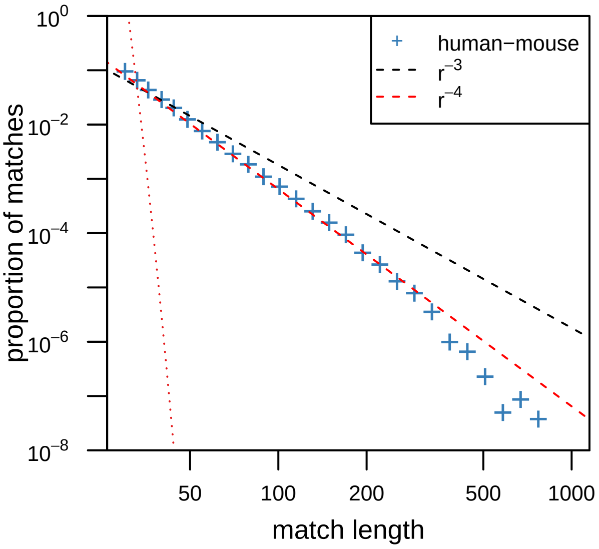

Figure S3: The MLD computed from the comparative alignment of the Human and Mouse genomes for unique matches only, see Materials and Methods for details. The MLD after filtering still exhibit a −4 power-law distribution.

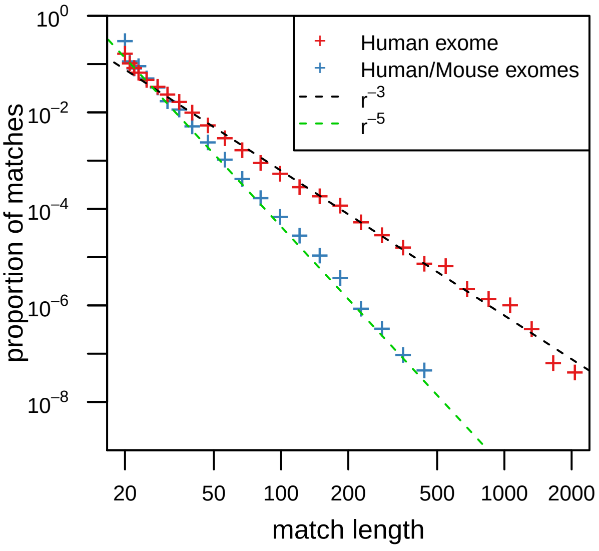

Figure S4: The MLD computed from a self-alignment of the Human exome (red) and from a comparative alignment of the exomes of Human and Mouse (blue). These exomes are obtained by concatenating all exons retrieved from the Ensembl database. In case two exons overlap, we merged them into one longer sequence containing the two exons. To separate the different non-overlapping exons, we added a letter 'N' between them. The dashed lines represent power-laws with exponent $\alpha = -3$ (black) and $\alpha = -5$ (green). Empirical data are represented using logarithmic binning to reduce the sampling noise.

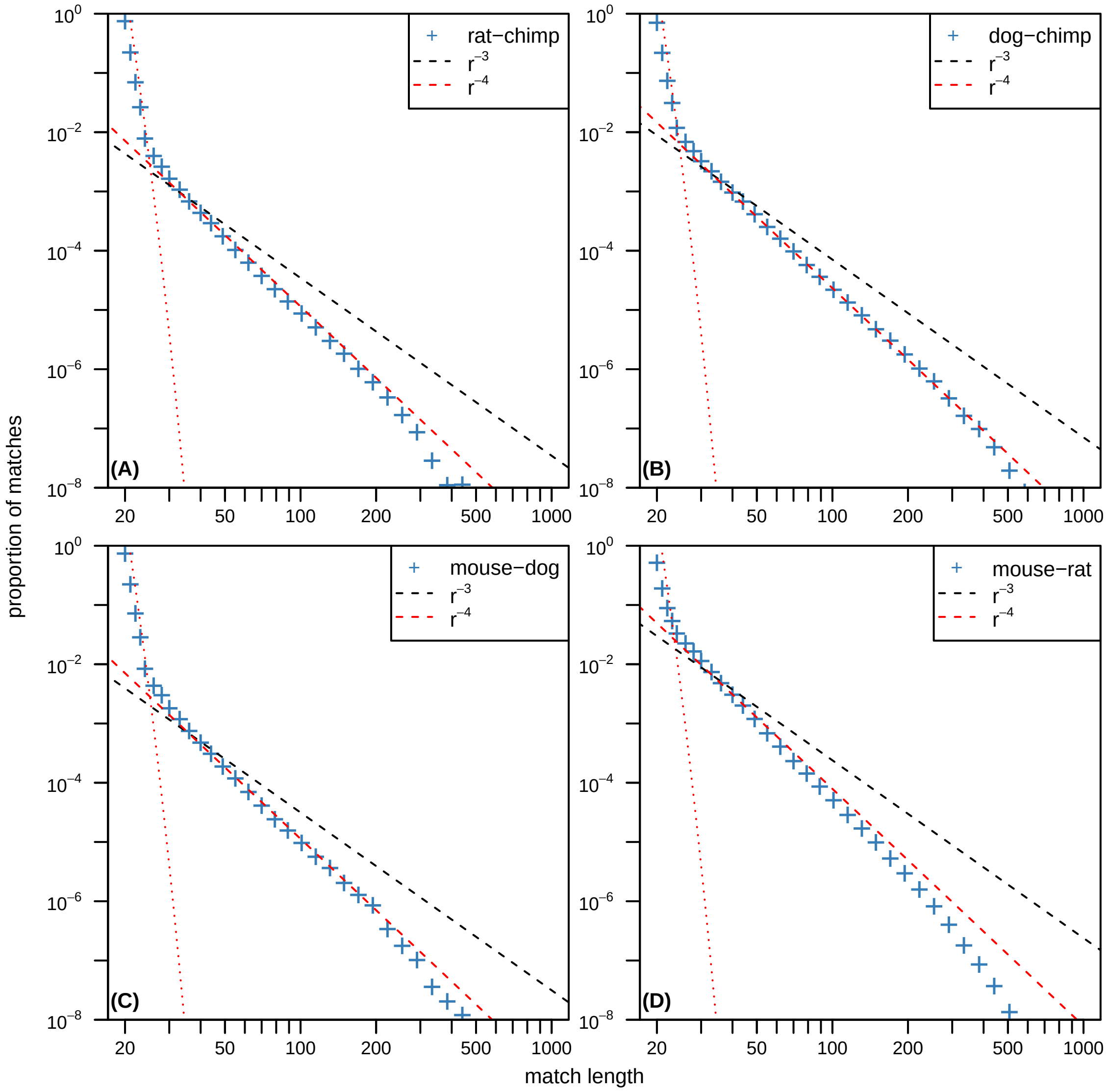

Figure S5: The MLD computed for the self-alignment of RepeatMasked genomes of different species. The dashed lines represent power-laws with exponent $alpha = -3$ and $\alpha = -4$. All the empirical data are represented using logarithmic binning to reduce the sampling noise. (A) The comparative alignment of Rat and Chimp genomes. (B) T The comparative alignment of Dog and Chimp genomes. (C) The comparative alignment of Mouse and Dog genomes. (D) The comparative alignment of Mouse and Rat genomes
